# Statistical biopsy: An emerging screening approach for early detection of cancers

**DOI:** 10.3389/frai.2022.1059093

**Published:** 2023-01-20

**Authors:** Gregory R. Hart, Vanessa Yan, Bradley J. Nartowt, David A. Roffman, Gigi Stark, Wazir Muhammad, Jun Deng

**Affiliations:** ^1^Institute for Disease Modeling, Global Health Division, Bill and Melinda Gates Foundation, Seattle, WA, United States; ^2^Department of Therapeutic Radiology, Yale University, New Haven, CT, United States; ^3^SMFE, Wright-Patterson Air Force Base, Dayton, OH, United States; ^4^Research Partners, Sun Nuclear Corporation (Mirion Technologies Inc.), Melbourne, FL, United States; ^5^Department of Physics, Florida Atlantic University, Boca Raton, FL, United States

**Keywords:** cancer screening, machine learning and AI, neural network, biopsy, data mining, cancer detection, individualized medicine

## Abstract

Despite large investment cancer continues to be a major source of mortality and morbidity throughout the world. Traditional methods of detection and diagnosis such as biopsy and imaging, tend to be expensive and have risks of complications. As data becomes more abundant and machine learning continues advancing, it is natural to ask how they can help solve some of these problems. In this paper we show that using a person's personal health data it is possible to predict their risk for a wide variety of cancers. We dub this process a “statistical biopsy.” Specifically, we train two neural networks, one predicting risk for 16 different cancer types in females and the other predicting risk for 15 different cancer types in males. The networks were trained as binary classifiers identifying individuals that were diagnosed with the different cancer types within 5 years of joining the PLOC trial. However, rather than use the binary output of the classifiers we show that the continuous output can instead be used as a cancer risk allowing a holistic look at an individual's cancer risks. We tested our multi-cancer model on the UK Biobank dataset showing that for most cancers the predictions generalized well and that looking at multiple cancer risks at once from personal health data is a possibility. While the statistical biopsy will not be able to replace traditional biopsies for diagnosing cancers, we hope there can be a shift of paradigm in how statistical models are used in cancer detection moving to something more powerful and more personalized than general population screening guidelines.

## Introduction

Cancer is a global public health burden with an estimated 21.7 million new cases and 13 million cancer deaths annually by 2030 (Ferlay et al., 2019). Despite a huge amount of money and resources spent on cancer screening, diagnosis, and treatment, it is estimated that 609,360 people in the United States will die from cancer in 2022

alone (Siegel et al., 2022). One important factor contributing to the high mortality is the lack of an efficient tool for cancer screening, missing the most effective window of opportunity for detecting cancers at their earliest stages. Another factor is the lack of individualized risk management for tailored cancer prevention. Hence, it is critical to develop safe and cost-effective approaches for cancer screening prior to disease onset with high sensitivity, specificity, and accessibility.

Tissue biopsy has long been used to diagnose cancer and often considered the gold standard, but it is limited by constraints on sampling frequency and incomplete representation of the organ being biopsied (Bravo et al., 2001). In addition, the surgical procedure is invasive, time-intensive, and costly with pain and risk of complications. Liquid biopsy offers a non-invasive alternative to cancer screening, but detection and analysis of circulating tumor DNA in a body fluid specimen present a considerable challenge (Alix-Panabières and Pantel, 2013; Crowley et al., 2013). Another challenge for liquid biopsy is how to identify the tumor site in the body, even after an individual has tested positive (Su, 2019).

Numerous schemas have been developed to improve clinical decision-making in cancer screening, detection, and prevention (Kramer, 2004; Holle, 2017).^1^^−^^3^ While cancer screening usually involves a procedure or body fluid test to detect cancer at an early stage, cancer prevention aims to reduce cancer risk and mortality by avoiding carcinogens, modifying lifestyles, and using chemoprevention (Kramer, 2004; Holle, 2017). As of now, routine cancer screening is only recommended for breast, cervical, colorectal, lung, and prostate cancers (see Footnotes 1–3). Cancer prevention strategies are only available for breast cancer, colorectal cancer, human papillomavirus-related cancers (anal, cervical, penile, vaginal, and vulvar cancers), ovarian cancer, and prostate cancer, as recommended by the American Cancer Society (ACS), National Comprehensive Cancer Network (NCCN), and US. Preventive Services Task Force (USPSTF) (see Footnotes 1–3). While the benefits of those schemas may include reduced cancer incidence and cancer mortality, their common limitations include the requirement of clinical testing, suboptimal positive/negative predictive values, frequent involvement of invasive procedures, and over diagnosis and overtreatment (Kramer, 2004; Holle, 2017). Ideally, it would be in the best interest of people to improve estimates of cancer risk prior to any clinical testing so that the cost and potential harms associated with invasive procedures would be limited (Cruz and Wishart, 2006; Ayer et al., 2010; Kourou et al., 2014; Boursi et al., 2017; Rajkomar et al., 2019).

**Figure 1:**
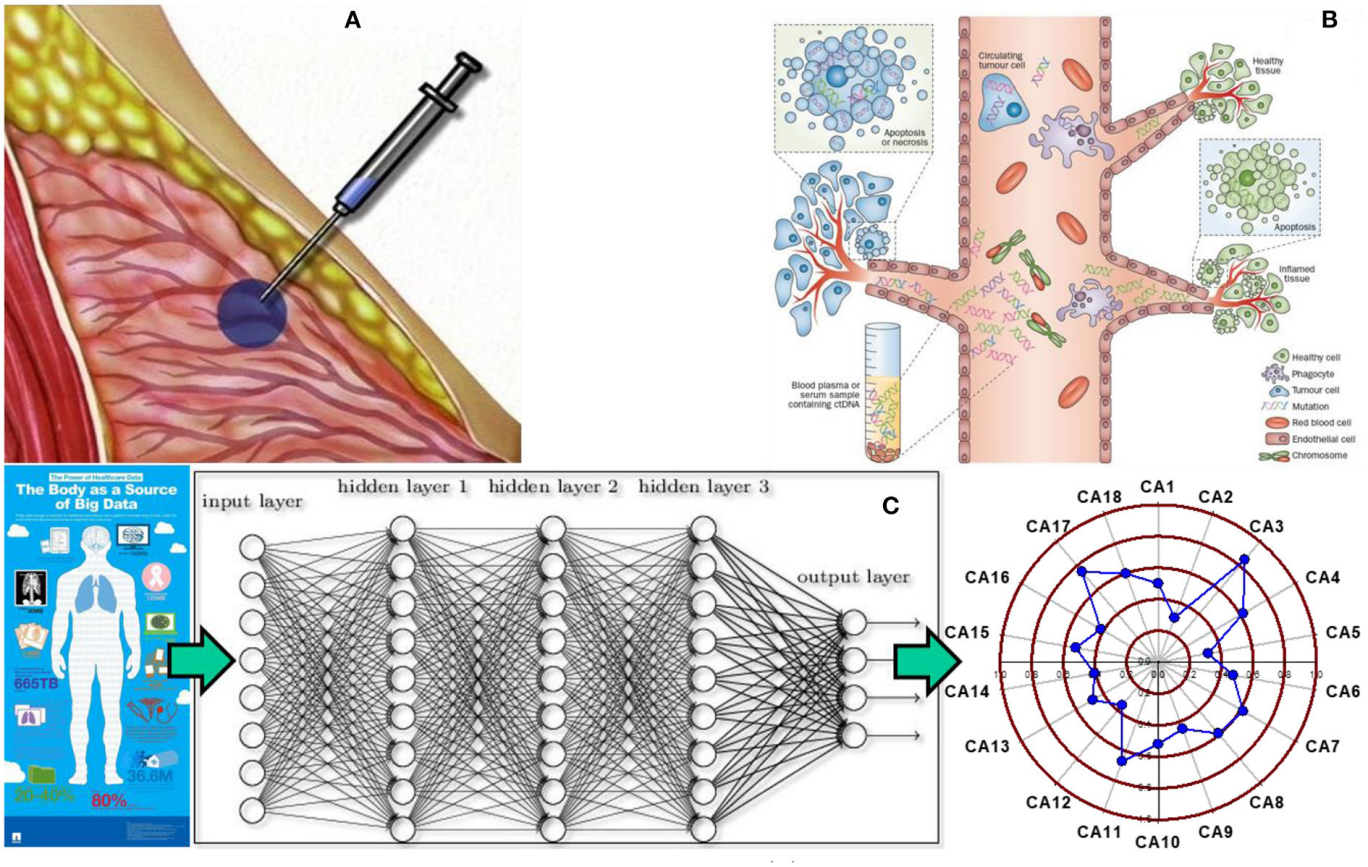
Tissue biopsy, liquid biopsy, and statistical biopsy **(A)** tissue biopsy is used to characterize tissues and diagnose cancer by evaluating the cells from a tissue specimen. However, it is an invasive, time intensive, and costly procedure, which inflicts pain and risk on patients. **(B)** Liquid biopsy has been recently developed to evaluate circulating tumor DNA from a body fluid sample to screen for cancer. It offers a noninvasive alternative to cancer screening, but detection and analysis of circulating tumor DNA in a body fluid specimen remains a challenging task for medical researchers and practitioners. **(C)** Statistical biopsy is a new approach proposed by our group that mines personal health data for early cancer detection with sophisticated statistical modeling. The basic idea is that a trove of personal health data can be used to train and validate deep learning models to generate a holistic profile of one's risks for a variety of cancers simultaneously prior to disease onset. Panel **(A)** adapted from the PreOp website (https://preop.com/wp-content/uploads/2021/08/333_surgery.jpg).

Recently, we have demonstrated that deep neural networks, trained and validated with the National Health Interview Survey (NHIS) and/or the Prostate, Lung, Colorectal, and Ovarian (PLCO) Cancer Screening Trial datasets, can be used to predict and stratify cancer risks with high discriminatory power based solely on personal health data (Hart et al., 2018, 2019, 2020; Roffman et al., 2018a,b; Muhammad et al., 2019; Nartowt et al., 2019a,b; Stark et al., 2019). Compared to the clinician's judgment, the strong performance of our models presents a novel opportunity to perform a “statistical biopsy” on individuals prior to disease onset (Hart et al., 2020). As shown in Figure 1, statistical biopsy mines personal health data from individuals for early cancer detection, analogous to tissue biopsy evaluating cells from a tissue specimen and liquid biopsy evaluating circulating tumor DNA from a fluid sample. What is different is that statistical biopsy seeks to decipher the invisible correlations and inter-connectivity between multiple medical conditions and health parameters *via* sophisticated statistical modeling. With statistical biopsy, it is possible to generate a holistic analysis of an individual's risk for a variety of cancers simultaneously. Furthermore, if integrated into a modern electronic medical record (EMR) system, it offers a cost-effective and safe approach to cancer screening in real time, informing preventive interventions and screening decisions.

In order to personalize early cancer detection and prevention, an accurate risk assessment of a variety of cancers for each individual is needed. Hence, we begin the development of a novel cancer risk profiler based on deep learning of personal health data for better risk stratification and more precise screening. We hypothesize that the trove of personal health data, including clinical and demographic data, family history, socio-behavioral, dietary and lifestyle data, can be used to train and validate a deep learning model capable of screening cancer prior to disease onset, with high sensitivity and specificity and with minimal toxicity and maximal accessibility.

## Materials and methods

### Data sets

In this work we use two large medical datasets, one for training a neural network to predict the appearance of cancer within 5 years and the other for testing the neural network. The first is the Prostate, Lung, Colorectal, and Ovarian (PLCO) trial (Tammemagi et al., 2011) which is used for training. The testing set came from the UK Biobank database (UK Biobank, 2022).

PLCO was a randomized controlled trial investigating the effectiveness of screening methods for prostate, lung, colorectal, and ovarian cancers. PLCO enrolled 154,897 participants 55–75 years of age between November 1993 and July 2001 in the United States. Participants were followed for 13 years, until they developed cancer, or passed away. We removed those that did not complete the baseline health survey leaving 149,623 participants. PLCO recorded the appearance of 13 general cancers (biliary, bladder, colorectal, glioma, head and neck, hematopoietic, liver, lung, melanoma, pancreas, renal, thyroid, and upper GI cancers), 3 female specific cancers (breast, endometrial, and ovarian), and 2 male specific cancers (male breast and prostate). In addition to these cancers, we use 116 general features, 20 female specific features, and 12 male specific features. We split the data into a set for females to predict 16 cancer types and set for males to predict 15 cancer types. See Table 1 for a list of features and their statistics and Table 2 for the number of cancer cases.

**Table 1:** Feature distributions and missingness.

**Feature**	**Female**		**Male**	
	**Train**	**Test**	**Train**	**Test**
**Binary**	**% Yes (% missing)**		**% Yes (% missing)**	
Ever had arthritis	45.82 (0.00)	2.62 (0.00)	29.97 (0.67)	3.80 (0.00)
Ever had chronic bronchitis	5.94 (0.00)	1.10 (76.49)	3.64 (0.68)	1.17 (75.35)
Ever had colon co-morbidity	1.70 (0.00)	1.32 (0.00)	1.17 (1.02)	1.14 (0.00)
Ever had diabetes	6.42 (0.00)	1.03 (0.59)	9.07 (0.63)	3.83 (0.43)
Ever had diverticulitis or diverticulosis	8.32 (0.00)	7.51 (0.00)	5.38 (0.78)	7.01 (0.00)
Ever had emphysema	2.05 (0.00)	0.40 (76.49)	3.05 (0.65)	0.22 (75.35)
Ever had gall bladder stones or inflammation	15.90 (0.00)	2.94 (0.00)	6.99 (0.73)	5.54 (0.00)
Ever had coronary heart disease or a heart attack	4.84 (0.00)	11.62 (0.00)	13.46 (0.64)	4.10 (0.00)
Ever had high blood pressure	33.97 (0.00)	26.16 (0.00)	34.38 (0.59)	19.39 (0.00)
Ever had liver co-morbidity	3.37 (0.00)	0.65 (0.00)	4.09 (0.77)	0.37 (0.00)
Ever had osteoporosis	9.64 (0.00)	0.62 (0.00)	0.82 (0.75)	1.93 (0.00)
Ever had colorectal polyps	5.54 (0.00)	6.08 (0.00)	8.12 (0.75)	4.04 (0.00)
Ever had a stroke	2.14 (0.00)	0.72 (0.00)	2.75 (0.63)	0.37 (0.00)
Ever smoked regularly	44.34 (0.00)	65.30 (0.58)	63.52 (0.03)	55.20 (0.52)
Current smoker	9.71 (0.00)	12.56 (0.60)	11.71 (0.03)	8.96 (0.53)
Family history of biliary cancer	0.34 (0.00)	– (100.00)	0.20 (4.50)	– (100.00)
Family history of bladder cancer	2.18 (0.00)	– (100.00)	1.51 (4.48)	– (100.00)
Family history of breast cancer	14.56 (0.00)	12.55 (23.42)	– (100.00)	12.97 (16.78)
Family history of colorectal cancer	11.33 (0.00)	14.14 (23.10)	9.29 (4.31)	12.57 (16.87)
Family history of endometrial cancer	2.89 (0.00)	– (100.00)	– (100.00)	– (100.00)
Family history of glioma cancer	2.01 (0.00)	– (100.00)	1.74 (4.46)	– (100.00)
Family history of head and neck cancer	1.42 (0.00)	– (100.00)	1.09 (4.48)	– (100.00)
Family history of hematopoietic cancer	6.67 (0.00)	– (100.00)	5.35 (4.40)	– (100.00)
Family history of liver cancer	2.04 (0.00)	– (100.00)	2.19 (4.44)	– (100.00)
Family history of lung cancer	11.71 (0.00)	15.14 (22.51)	9.85 (4.28)	14.69 (16.34)
Family history of male breast cancer	– (100.00)	– (100.00)	21.01 (2.47)	– (100.00)
Family history of melanoma cancer	1.40 (0.00)	– (100.00)	0.80 (4.49)	– (100.00)
Family history of ovarian cancer	3.93 (0.00)	– (100.00)	– (100.00)	– (100.00)
Family history of pancreas cancer	3.06 (0.00)	– (100.00)	2.18 (4.47)	– (100.00)
Family history of prostate cancer	– (100.00)	– (100.00)	7.40 (2.53)	9.65 (17.15)
Family history of renal cancer	1.79 (0.00)	– (100.00)	1.25 (4.48)	– (100.00)
Family history of thyroid cancer	0.70 (0.00)	– (100.00)	0.35 (4.50)	– (100.00)
Family history of upper GI cancer	4.51 (0.00)	– (100.00)	4.63 (4.41)	– (100.00)
Ever had enlarged prostate			21.80 (0.18)	0.00 (0.00)
Ever had inflamed prostate			8.45 (16.54)	0.00 (0.00)
Ever had a prostate biopsy			4.98 (2.90)	0.00 (0.00)
Ever had a prostatectomy			0.31 (3.21)	– (100.0)
Ever had a prostate resection			2.98 (3.16)	0.00 (0.00)
Ever had a vasectomy			27.28 (0.35)	0.00 (0.00)
Had ovaries removed	16.57 (0.00)			
Had tubes tied	21.49 (0.00)	0.00 (0.00)		
Ever take birth control pills	54.22 (0.00)	– (100.00)		
Currently using female hormones	49.33 (0.00)	0.00 (0.00)		
Ever take female hormones	66.37 (0.00)	– (100.00)		
Ever been pregnant	92.49 (0.00)	– (100.00)		
Ever dealt with infertility	14.51 (0.00)	0.00 (0.00)		
Ever had benign or fibrocystic breast disease	28.45 (0.00)	0.01 (0.00)		
Ever had benign ovarian tumor/cyst	12.80 (0.00)	0.00 (0.00)		
Ever had endometriosis	8.39 (0.00)	0.00 (0.00)		
Ever had Uterine fibroid tumors	22.48 (0.00)	0.00 (0.00)		
**Categorical**	**% in Category**		**% in Category**	
**Race**				
White	88.55	93.97	88.37	94.18
Black	5.68	1.65	4.56	1.96
Hispanic	1.60	0.00	2.17	0.00
Asian	3.37	2.72	4.07	2.22
Pacific Islander	0.49	0.00	0.62	0.00
American Indian	0.27	0.00	0.25	0.00
Missing	0.04	1.66	0.06	1.64
**Education level**				
< 8 years	0.72	0.00	1.25	0.00
8–11 years	5.82	0.00	7.00	0.00
12 years	27.47	23.85	18.25	28.55
Non-college training	12.85	4.48	12.25	5.76
Some college	23.15	19.28	20.41	16.24
College graduate	15.02	33.56	18.83	31.02
Postgraduate	14.71	0.0	21.73	0.00
Missing	0.26	18.8	0.29	18.44
**Marriage status**				
Married or cohabitating	68.71	76.73	82.51	69.47
Widowed	13.85	0.00	3.60	0.00
Divorced	12.91	0.00	9.05	0.00
Separated	0.92	0.00	1.11	0.00
Never married	3.39	0.00	3.43	0.00
Missing	0.23	23.27	0.29	30.53
**Occupation**				
Homemaker	22.23	0.54	0.08	4.62
Working	35.18	60.23	44.00	54.59
Unemployed	0.96	2.35	1.16	1.06
Retired	36.58	31.22	49.59	35.00
Extended sick leave	0.20	0.00	0.17	0.00
Disabled	2.08	4.09	2.42	2.73
Other	2.24	0.89	2.09	0.94
Missing	0.52	1.08	0.49	1.06
**Continuous**	**Mean (SD); % missing**		**Mean (SD); % missing**	
Age at enrollment	62.5 (5.4); 0.0	56.7 (8.2); 0.0	62.7 (5.3); 0.0	56.3 (8.0); 0.0
BMI at enrollment	27.1 (5.5); 0.0	27.8 (4.2); 0.0	27.5 (4.2); 1.6	27.1 (5.2); 0.5
Weight at age 20	124 (18.1); 0.0	– (–); 100.0	160 (24.3); 1.3	– (–); 100.0
Years since quitting smoking	25.0 (13.3); 0.0	24.6 (14.2); 1.0	16.9 (13.5); 1.0	25.3 (17.8); 46.0
Pack years smoked	13.3 (22.4); 0.0	26.1 (20.9); 1.0	25.2 (31.5); 2.3	20.2 (15.5); 51.4
Monthly aspirin use	9.8 (16.5); 0.0	0.0 (0.0); 0.0	12.2 (16.7); 0.3	0.0 (0.0); 0.0
Monthly ibuprofen use	7.5 (17.4); 0.0	0.0 (0.0); 0.0	4.9 (14.3); 0.5	0.0 (0.0); 0.0
Youngest relative with biliary cancer	68.1 (5.4); 0.0	– (–); 100.0	68.4 (12.2); 0.0	– (–); 100.0
Youngest relative with bladder cancer	67.7 (6.3); 0.0	– (–); 100.0	67.9 (11.9); 1.9	– (–); 100.0
Youngest relative with breast cancer	58.4 (8.0); 0.0	– (–); 100.0	– (–); 0.0	– (–); 100.0
Youngest relative with colorectal cancer	66.2 (6.8); 0.0	– (–); 100.0	65.7 (12.7); 2.1	– (–); 100.0
Youngest relative with endometrial cancer	56.0 (7.2); 0.0	– (–); 100.0	– (–); 0.0	– (–); 100.0
Youngest relative with glioma cancer	54.9 (8.3); 0.0	– (–); 100.0	55.0 (17.8); 1.3	– (–); 100.0
Youngest relative with head and neck cancer	60.7 (5.6); 0.0	– (–); 100.0	61.4 (13.0); 2.6	– (–); 100.0
Youngest relative with hematopoietic cancer	57.0 (10.1); 0.0	– (–); 100.0	56.1 (20.0); 1.9	– (–); 100.0
Youngest relative with liver cancer	64.2 (6.6); 0.0	– (–); 100.0	65.3 (12.5); 1.6	– (–); 100.0
Youngest relative with lung cancer	65.0 (6.1); 0.0	– (–); 100.0	63.9 (11.5); 1.7	– (–); 100.0
Youngest relative with male breast cancer	– (–); 0.0	– (–); 100.0	58.8 (15.6); 2.4	– (–); 100.0
Youngest relative with melanoma cancer	55.9 (8.9); 0.0	– (–); 100.0	56.8 (17.3); 1.2	– (–); 100.0
Youngest relative with ovarian cancer	57.9 (8.1); 0.0	– (–); 100.0	– (–); 0.0	– (–); 100.0
Youngest relative with pancreas cancer	68.9 (5.7); 0.0	– (–); 100.0	67.9 (11.9); 1.0	– (–); 100.0
Youngest relative with prostate cancer	– (–); 0.0	– (–); 100.0	70.3 (9.8); 2.5	– (–); 100.0
Youngest relative with renal cancer	63.1 (6.7); 0.0	– (–); 100.0	62.5 (14.9); 2.4	– (–); 100.0
Youngest relative with thyroid cancer	43.4 (8.3); 0.0	– (–); 100.0	49.2 (18.6); 2.9	– (–); 100.0
Youngest relative with upper GI cancer	64.3 (6.7); 0.0	– (–); 100.0	63.6 (13.8); 2.0	– (–); 100.0
Number of relatives with biliary cancer	1.0 (0.2); 0.0	– (–); 100.0	1.0 (0.1); 0.0	– (–); 100.0
Number of relatives with bladder cancer	1.0 (0.2); 0.0	– (–); 100.0	1.0 (0.2); 0.0	– (–); 100.0
Number of relatives with breast cancer	1.1 (0.3); 0.0	1.0 (0.0); 0.0	– (–); 0.0	1.0 (0.0); 0.0
Number of relatives with colorectal cancer	1.1 (0.3); 0.0	1.0 (0.0); 0.0	1.1 (0.3); 0.0	1.0 (0.0); 0.0
Number of relatives with endometrial cancer	1.0 (0.2); 0.0	– (–); 100.0	– (–); 0.0	– (–); 100.0
Number of relatives with glioma cancer	1.0 (0.2); 0.0	– (–); 100.0	1.0 (0.2); 0.0	– (–); 100.0
Number of relatives with head and neck cancer	1.0 (0.2); 0.0	– (–); 100.0	1.0 (0.2); 0.0	– (–); 100.0
Number of relatives with hematopoietic cancer	1.1 (0.3); 0.0	– (–); 100.0	1.1 (0.2); 0.0	– (–); 100.0
Number of relatives with liver cancer	1.0 (0.2); 0.0	– (–); 100.0	1.0 (0.2); 0.0	– (–); 100.0
Number of relatives with lung cancer	1.1 (0.4); 0.0	1.0 (0.0); 0.0	1.1 (0.3); 0.0	1.0 (0.0); 0.0
Number of relatives with male breast cancer	– (–); 0.0	– (–); 100.0	1.0 (0.1); 0.0	– (–); 100.0
Number of relatives with melanoma cancer	1.0 (0.2); 0.0	– (–); 100.0	1.0 (0.2); 0.0	– (–); 100.0
Number of relatives with ovarian cancer	1.0 (0.2); 0.0	– (–); 100.0	– (–); 0.0	– (–); 100.0
Number of relatives with pancreas cancer	1.0 (0.2); 0.0	– (–); 100.0	1.0 (0.2); 0.0	– (–); 100.0
Number of relatives with prostate cancer	– (–); 0.0	– (–); 100.0	1.1 (0.3); 0.0	1.0 (0.0); 0.0
Number of relatives with renal cancer	1.0 (0.2); 0.0	– (–); 100.0	1.0 (0.1); 0.0	– (–); 100.0
Number of relatives with thyroid cancer	1.0 (0.2); 0.0	– (–); 100.0	1.0 (0.2); 0.0	– (–); 100.0
Number of relatives with upper GI cancer	1.0 (0.2); 0.0	– (–); 100.0	1.1 (0.3); 0.0	– (–); 100.0
Age when prostate became enlarged			52.6 (9.3); 0.5	56.8 (10.2); 0.0
Age when prostate became inflamed			45.0 (13.2); 0.6	– (–); 0.0
How many times you get up at night to urinate			1.3 (0.9); 0.2	– (–); 100.0
Age at which you started urinating at night			50.5 (10.5); 58.5	– (–); 0.0
Age at first prostate surgery			54.9 (7.9); 7.4	54.5 (6.9); 0.0
Age at vasectomy			29.0 (3.5); 0.5	– (–); 0.0
Age at hysterectomy	41.5 (4.6); 0.0	– (–); 100.0		
Age started birth control	24.8 (6.4); 0.0	– (–); 100.0		
Number of years taking female hormones	6.8 (3.0); 0.0	– (–); 100.0		
Age at birth of first child	21.0 (4.5); 0.0	– (–); 100.0		
Number of live births	3.1 (1.3); 0.0	– (–); 100.0		
Number of miscarriages	0.5 (0.7); 0.0	– (–); 100.0		
Number of still births	0.1 (0.3); 0.0	– (–); 100.0		
Number of tubal/ectopic pregnancies	0.0 (0.2); 0.0	– (–); 100.0		
Age at first menstrual period	12.2 (1.6); 0.0	– (–); 100.0		

**Table 2:** Count of cancer cases in the data sets.

**Cancer**	**Female**		**Male**	
	**Train**	**Test**	**Train**	**Test**
Biliary	20	77	10	53
Bladder	89	276	387	781
Breast	1,912	4,525	13	31
Colorectal	429	1,034	681	1,352
Endometrial	352	614	–	–
Glioma	42	452	60	459
Head and Neck	63	264	171	681
Hematopoietic	351	651	482	849
Liver	8	1,082	60	1,050
Lung	526	949	806	838
Melanoma	195	599	289	575
Ovarian	225	514	–	–
Pancreas	89	208	134	202
Prostate	–	–	3,749	3,365
Renal	98	229	155	407
Thyroid	47	118	31	46
Upper GI	30	160	164	338

UK Biobank is a large-scale biomedical database trying to accelerate medical and public health research by gathering and maintaining a staggering amount of information. They enrolled half a million participants from 2006 to 2010. Many types of follow-up and additions are frequently made. Everything from repeating the baseline health evaluation to imaging and sequencing. Information is pulled from death and cancer registries and hospital admissions and primary care data. From this data base we have 229,263 male participants and 273,375 female participants. The UK Biobank data is more detailed than the PLCO data, so we map it onto the PLCO features we used in training.

**Figure 2:**
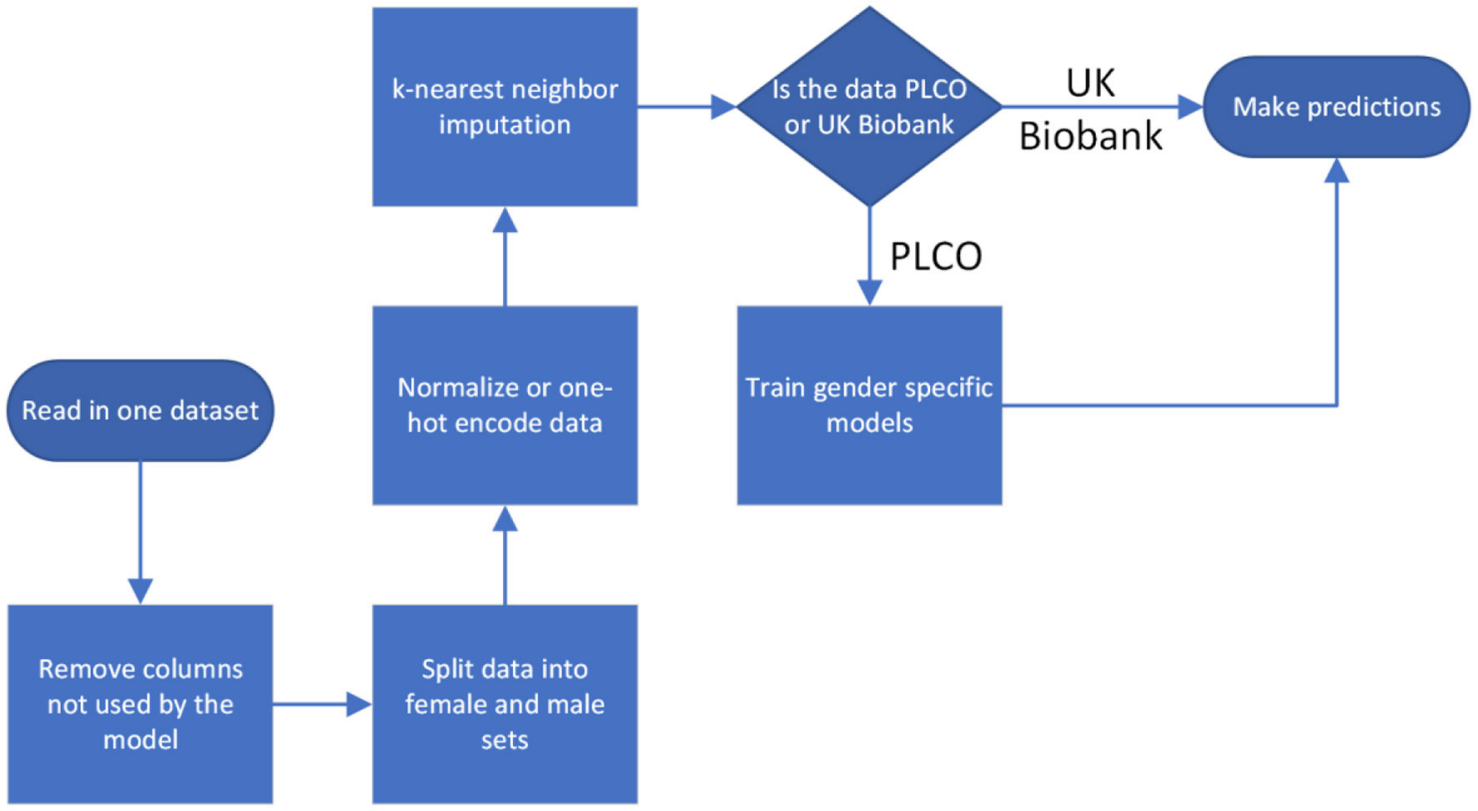
Flow process for data preparation and model training.

For both datasets we normalized all the inputs, situating them within the range 0–1. Categorical inputs were handled using one-hot encoding. For the cancer diagnoses we considered diagnoses <5 years after baseline evaluation to be positive and all others to be negative. We handled missing data through *k*-nearest neighbor imputation with *k* = 5. Imputation was done separately on PLCO and UK Biobank so that there was no information passed between them, except in the case of a feature completely missing from UK Biobank, in which case we set it to the mean value from the PLCO dataset (Figure 2).

The data was read in and processed in Python with the Pandas library, version 1.5.1. The Pandas data frames were converted to 2d Numpy arrays (version 1.23.4) before being passed to the training software.

### Neural network

Using the PLCO dataset we train two different neural networks, one to take in the female data and predict the risk for 16 different cancers and another to take in the male data and predict the risk for 15 different cancers. The networks were trained as binary classifiers, with the positive class being those that developed cancer within 5 years of enrolling in the study. Each network has 2 hidden layers with 120 nodes in the first layer and 80 in the second. This network architecture was chosen because it was previously used with good results in a master's thesis that used the PLCO dataset to predict cancer risk (Yan, 2020). For both the female and male models the biases are initialized to 0 and weights are initialized with a glorot normal initializer. We used the ReLu activation function and the Adam optimizer with a learning rate of 0.01. To avoid the exploding gradient problem, we use gradient clipping. For the loss function we use binary cross-entropy. We train with batch sizes of 1,024 for 10 epochs. The prediction for each cancer coming from the output layer was put through a logistic function to scale it to the interval 0–1. We think of these values as the probability of developing cancer and later will multiply them by 100 and use them as the percent risk of developing cancer. The training and predictions were done with TensorFlow 2 *via* Keras, version 2.11.0.

For each cancer the neural network returns a number in the range of 0–1. Traditionally a threshold value of 0.5 is selected so that values ≥0.5 are considered positive and values below 0.5 are considered negative. However, in the data we are using there are more people without cancer than with cancer. This data imbalance can lead to bias in the predictions, but this can be addressed by avoiding the default threshold value. We empirically set the threshold (for each cancer) to maximize the Youden index. The Youden index is the difference between the true positive rate and the false positive rate. Maximizing this index picks the threshold value where the ROC curve begins to bend. We maximize the Youden index using the training data and then apply the results thresholds to the testing data (Duda et al., 2001; Bishop, 2006; Mitchell, 2006).

## Results

**Figure 3:**
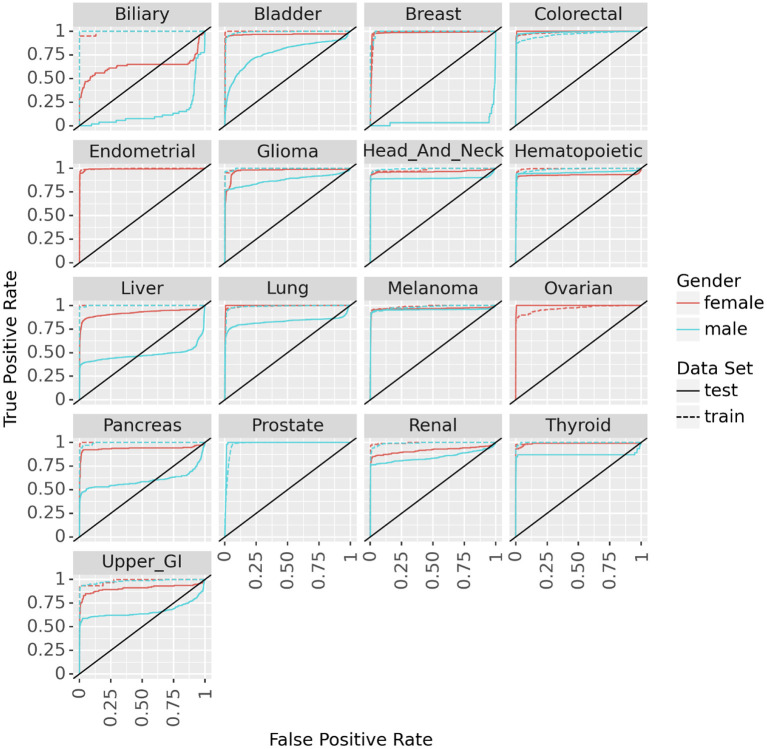
ROC curves for the neural networks trained on PLCO data and tested on UK Biobank data to predict 17 different cancers.

Fitting the neural network to predict cancer incidence within 5 years for all 17 cancer types is quite successful. Looking at the ROC for the PLCO data (dotted lines in Figure 3) the classifier is near perfect for every cancer. This is further confirmed by looking at various metrics of effectiveness. On this training data no cancer has an AUC below 0.98, informedness below 0.85, or diagnostic odds ratio below 270 (see Table 3).

**Table 3:** Metrics of performance.

	**Cutoff**	**Positive predictive value**	**Negative predictive value**	**AUC of ROC**	**Matthews correlation coefficient**	**Informed-ness**	**Diagnostic odds ratio**
**Biliary**							
Female							
Train	0.263	0.6129	1.0000	0.9933	0.7630	0.9498	120,276
Test		0.0004	0.9998	0.6341	0.0061	0.1804	7
Male							
Train	0.028	0.3448	1.0000	0.9999	0.5871	0.9997	Inf
Test		Nan	1.0000	0.1339	Nan	0.0000	0.0000
**Bladder**							
Female							
Train	0.002	0.2145	1.0000	0.9995	0.4621	0.9957	Inf
Test		0.1047	0.9999	0.9658	0.3113	0.9264	1,727
Male							
Train	0.002	0.1414	0.9997	0.9911	0.3609	0.9229	691
Test		0.0139	0.9984	0.7727	0.0750	0.4569	12
**Breast**							
Female							
Train	0.025	0.4344	0.9997	0.9883	0.6443	0.9563	2,942
Test		0.0391	0.9996	0.9815	0.1498	0.5788	319
Male							
Train	0.001	0.0044	1.000	0.9950	0.0653	0.9605	Inf
Test		0.0000	0.9999	0.3992	−0.0018	−0.0242	0
**Colorectal**							
Female							
Train	0.003	0.2396	0.9997	0.9860	0.4734	0.9362	1,211
Test		0.0062	1.0000	0.9979	0.0496	0.3948	Inf
Male							
Train	0.006	0.2273	0.9989	0.9640	0.4415	0.8616	295
Test		0.441	0.9999	0.9887	0.1938	0.8546	463
Female							
Train	0.003	0.2892	0.9999	0.9954	0.5292	0.9689	4,445
Test		0.0724	0.9999	0.9911	0.2589	0.9266	775
**Glioma**							
Female							
Train	0.284	0.4082	1.0000	0.9959	0.6232	0.9516	26,203
Test		0.0686	0.9984	0.9732	0.0416	0.0259	40
Male							
Train	0.009	0.3333	1.0000	0.9972	0.5672	0.9651	18,427
Test		0.5401	0.9987	0.8773	0.4275	0.3393	893
**Head and Neck**							
Female							
Train	0.001	0.0432	1.0000	0.9858	0.2026	0.9505	1,745
Test		0.0287	0.9999	0.9660	0.1615	0.9112	536
Male							
Train	0.003	0.0839	0.9999	0.9948	0.2816	0.9461	1,380
Test		0.3362	0.9996	0.8963	0.5395	0.8668	1,291
**Hematopoietic**							
Female							
Train	0.005	0.1424	0.9999	0.9945	0.3683	0.9527	1,845
Test		0.0024	0.9981	0.9339	0.0029	0.0162	187
Male							
Train	0.011	0.2616	0.9996	0.9864	0.4897	0.9183	851
Test		0.4614	0.9997	0.9558	0.6461	0.9054	2,555
Female							
Train	0.043	0.3200	1.0000	0.9999	0.5656	0.9998	Inf
Test		0.5537	0.9981	0.9208	0.5321	0.5131	642
Male							
Train	0.291	0.4836	1.0000	0.9989	0.6893	0.9825	68,978
Test		0.0000	0.9954	0.4788	−0.0001	−0.0001	218
**Lung**							
Female							
Train	0.004	0.2603	0.9998	0.9902	0.4972	0.9504	1,692
Test		0.0625	1.0000	0.9981	0.2434	0.9471	Inf
Male							
Train	0.007	0.2978	0.9995	0.9878	0.5255	0.9292	856
Test		0.0644	0.9991	0.8314	0.2125	0.7114	78
**Melanoma**							
Female							
Train	0.002	0.2345	0.9999	0.9887	0.4695	0.9407	2,340
Test		0.0023	0.9991	0.9648	0.0061	0.0271	1,231
Male							
Train	0.013	0.3305	0.9997	0.9818	0.5522	0.9234	1,824
Test		0.7500	0.9975	0.9543	0.1249	0.0209	4,874
**Ovarian**							
Female							
Train	0.001	0.0733	0.9997	0.9681	0.2511	0.8641	270
Test		0.0022	1.0000	0.9989	0.0174	0.1383	Inf
**Pancreas**							
Female							
Train	0.003	0.2733	1.0000	0.9992	0.5190	0.9857	28,626
Test		0.0658	0.9998	0.9372	0.2262	0.7797	429
Male							
Train	0.002	0.0627	0.9999	0.9953	0.2430	0.2430	1,262
Test		0.3000	0.9991	0.5980	0.0665	0.0148	314
**Prostate**							
Male							
Train	0.040	0.4559	0.9992	0.9812	0.6478	0.9223	1,137
Test		0.3226	1.0000	0.9923	0.5589	0.9685	Inf
**Renal**							
Female							
Train	0.011	0.3862	1.0000	0.9957	0.6112	0.9674	15,944
Test		0.2194	0.9998	0.9170	0.4027	0.7401	1,302
Male							
Train	0.005	0.1014	0.9999	0.9921	0.3059	0.9243	938
Test		0.4878	0.9995	0.8460	0.5978	0.7333	2,001
**Thyroid**							
Female							
Train	0.314	0.4423	1.0000	0.9977	0.6577	0.9780	60,262
Test		0.5674	0.9999	0.9877	0.6967	0.8556	20,820
Male							
Train	0.001	0.0465	1.0000	0.9982	0.2112	0.9594	3,621
Test		0.0002	0.9999	0.8714	0.0048	0.1508	33
**Upper GI**							
Female							
Train	0.120	0.3011	1.0000	0.9845	0.5298	0.9325	16,371
Test		0.6000	0.9996	0.9078	0.3871	0.2499	3,503
Male							
Train	0.001	0.1629	0.9998	0.9329	0.3875	0.9222	1,314
Test		0.6238	0.9988	0.6655	0.3405	0.1862	1,345

We tested the model's generalizability on the UK Biobank data. Figure 3 (solid lines) shows that for most cancers the generalization is very good. The cancers that did not generalize well, biliary, male breast, liver, and pancreas, are those with the fewest cases in the training set and tend to have few cases in the test set as well (see Table 2). Also, the difference in the ROC curves tend to be larger for the model predicting cancer in males than for the one predicting cancer in females, indicating that the model for females generalized better than the model for males. Also, the male model did not generalize as well as the female model. However, the model this performs very well in terms of AUC and diagnostic odds ratio, with all but 3 cancers have diagnostic odds ratio above 10 with most of them still in the hundreds or thousands.

**Figure 4:**
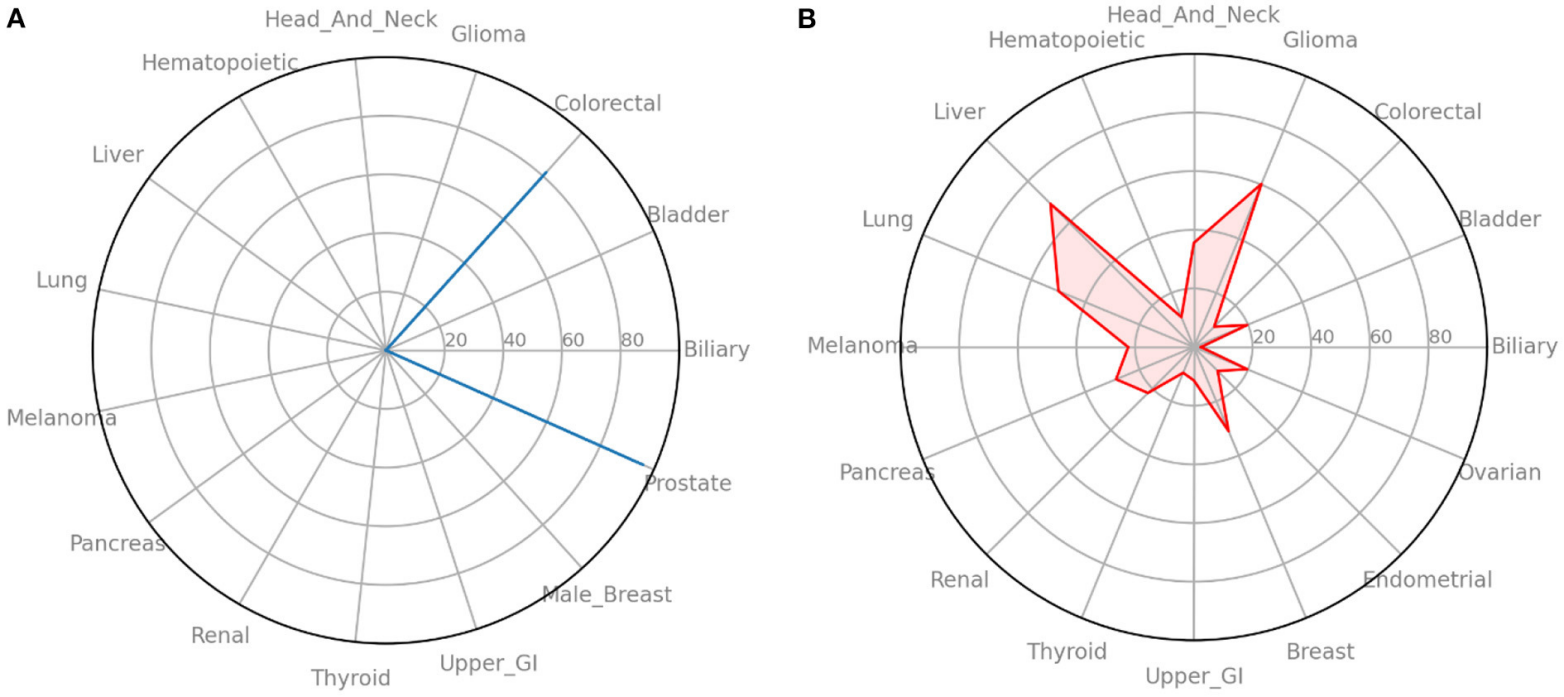
Radar plots of cancer risk for a single patient. **(A)** A male participant from the UK Biobank data that has high risk for two cancers. **(B)** A female participant from the UK Biobank data that has moderate risk for many cancers. Information such as this could help individuals and their primary care providers make decisions on screening and preventative measures.

In addition to simply training the neural network to predict future cancer incidence. We take the raw output of the model (always in the range of 0–1) as a risk indicator. Multiplying this risk by 100, we can treat it as a risk score and look at individual's risks across all cancers. In Figure 4A we see an example of such an analysis for a male from the UK Biobank dataset. It shows that he has high risk for colorectal and prostate cancer, but essentially no risks for the other cancers. While in Figure 4B we ran the same analysis for a female from the UK Biobank dataset and find that she has moderate risk for most cancers.

## Discussions

In this work we introduce the idea of a statistical biopsy, which mines personal health data from individuals for early cancer detection, analogous to tissue biopsy evaluating cells from a tissue specimen and liquid biopsy evaluating circulating tumor DNA from a fluid sample. Taking advantage of two rich datasets, PLCO and UK Biobank, we were able to train two neural networks (one for men and one for women) to predict cancer risk for 17 different cancers. This model was trained on the cancer focused PLCO dataset and then tested on the much larger UK Biobank dataset.

Testing with the UK Biobank dataset helps to show the model's generalizability and give us confidence that we are not overfitting the PLCO data, especially given the large number of features that we are using. Also given that the UK Biobank data comes from a different population, does not record all data in the same way, and is missing some of the features we used in our model, high performance on this dataset shows that the model has a high degree of robustness. Furthermore, the UK Biobank dataset is representative of the noisy and messy data that a physician would have access to *via* electronic medical records as opposed to much cleaner data gathered in a clinical trial, giving confidence that this idea can work in practice. While testing on this second dataset that comes from a different population adds a lot of confidence in the generalization of the model, it is important to note that both the training set and test set come from primarily Caucasian populations living in wealthy countries. Validating on additional datasets coming from other countries is important, especially depending on where this model is used.

Despite all this there were places where the model did not perform well. On cancers such as biliary, liver, and male breast cancer the model did not generalize at all and for two of these would do better if its predictions were reversed. Furthermore, on almost every cancer the male model generalized worst then the female model. This is particularly surprising since there are more missing female only features in the test set then in there are missing male only features. We need to further test the importance of this female/male only features and where there are other features that should be included. In addition to exploring feature importance, we are also working on quantifying the uncertainty in our prediction from these missing features and a way for the model to not only give a prediction but indicate which feature to learn to most improve the prediction. Also, while the diagnostic odds ratio is high for almost all the cancers, they need to be compared against tested screening guidelines (whether recommended or not) to see if our statistical biopsy is actually an improvement over traditional methods.

Lastly, while the stochastic nature of the development of cancer means a statistical biopsy could never completely replace a liquid or tissue biopsy, like the screening guidelines (see Footnote 1–3) it could point those traditional biopsies to individuals who would get the most benefit from them. Furthermore, it is possible to generate a holistic analysis of an individual's risk for a variety of cancers simultaneously, having the benefit of a liquid biopsy's general screening but retaining the specificity of a tissue biopsy (i.e., identifying which cancers one is at high risk for). Furthermore, if integrated into a modern electronic medical record (EMR) system, it offers a cost-effective and safe approach to cancer screening in real time, informing preventive interventions and screening decisions.

This model will form the backbone of a user-facing mobile health platform that will not only let individuals evaluate their cancer risk in real time, but also see the effect of certain preventative measures or lifestyle changes on those risks.

In the short term we hope that this mobile health platform will not only help individuals in early cancer detection, but also continue improving itself as it builds up a large and diverse longitudinal data set shared by the consented individuals.

Ultimately, we envision a model like this will be integrated into EMR systems, where every time an individual visits their doctor, has a test done, etc. it can update its predictions. It would assist physicians and patients, prompting conversations about cancer prevention and screenings as needed. In addition, as the model matures with more data, it could also provide information on what tests or diagnostics would provide the most information on cancer risk as well as the timing and spacing of such diagnostics.

While there are still many hurdles to overcome, at the scientific, social, and legal levels, there is already a good start toward this vision of statistical biopsies. Keeping active discussions on all three levels in the community is necessary for stakeholders to make steady progress toward the vision of statistical biopsy.

## Conclusion

We trained two neural networks to predict the risk of 16 types of cancers in females and 15 types in males and validated it against a second dataset that came from a different population. We showed this model could be used to look holistically at an individual's cancer risks. We introduced the term “statistical biopsy” to help change the paradigm around these types of models. With the large amounts of data available and powerful computers and algorithms it is time we move beyond guidelines for general population screening to more powerful and personalized methods akin to the liquid and tissues biopsies currently used in the medical field.

## Data availability statement

The existing datasets analyzed in this study can be accessed by application *via* the following links: https://cdas.cancer.gov/datasets/plco/ and https://www.ukbiobank.ac.uk/enable-your-research/apply-for-access.

## Author contributions

GH and VY developed models and code. GH, VY, and JD developed the core ideas and did most of the writing. BN, GH, VY, DR, GS, and WM did preliminary work predicting individual cancers with different models and datasets. All authors contributed to the article and approved the submitted version.

## Footnotes

^1^American Cancer Society Prevention and Early Detection Guidelines. https://www.cancer.org/health-care-professionals/american-cancer-society-prevention-early-detection-guidelines.html.

^3^United States Preventive Services Task Force Published Recommendations. https://www.uspreventiveservicestaskforce.org/BrowseRec/Index.
